# The effects of mulberry (*Morus alba* Linn.) leaf supplementation on growth performance, blood parameter, and antioxidant status of broiler chickens under high stocking density

**DOI:** 10.14202/vetworld.2022.2715-2724

**Published:** 2022-11-28

**Authors:** Charinya So-In, Nuchsupha Sunthamala

**Affiliations:** 1 Department of Veterinary Technology, Faculty of Agricultural Technology, Kalasin University, Kalasin 46000, Thailand; 2 Department of Biology, Faculty of Science, Mahasarakham University, Mahasarakham 44150, Thailand

**Keywords:** antioxidant, broiler chickens, growth performance, high stocking density, *Morus alba*, mulberry leaf

## Abstract

**Background and Aim:**

A stocking density system in boilers is well known for increasing productivity. However, this system increases stress and affects the growth performance of broilers. Mulberry is a valuable plant with therapeutic applications in traditional medicine; moreover, it reduces free radicals and improves growth performance in broilers. This study was conducted to investigate the effects of mulberry on the blood biochemistry parameters and the antioxidant status of broilers exposed to various raising systems.

**Materials and Methods:**

Two hundred and seventy-six 3-week-old male broilers were randomly assigned to nine categories composed of three growing systems: Semi-intensive, low stocking density, and high stocking density. Each group was fed with a control diet mixed with and without 10% mulberry leaf extract; the positive control group was provided with vitamin C. During the study, phytochemical screening of mulberry leaf extract, growth performances, hematological parameters, and antioxidant profiles were measured over the 4 weeks of the treatment.

**Results:**

In the high stocking density group, lipid peroxidation gradually increased while antioxidant activities decreased; however, the level of lipid peroxidation was reduced, whereas catalase and superoxide dismutase activities were significantly increased. The growth performance and blood biochemistry were improved after being fed with 10% mulberry leaf extract.

**Conclusion:**

This finding indicates that mulberry leaf extract reduced oxidative stress, activated antioxidant enzyme activities, and enhanced broilers’ growth performance when raised under stress conditions.

## Introduction

Among the different environmental factors resulting in stress, stocking density is considered important in terms of its influence on broiler production. It impacts broiler health, welfare, well-being, and performance [1]. Crowded living spaces can positively impact farmer incomes, which generally increase stocking density [2]. However, a high stocking density can be stressful and has deleterious effects on broiler performance and immunity [3]. Many studies have indicated that broiler health and welfare can be achieved at a range of densities, most likely varying between 34 and 38 kg/m^2^ [4, 5].

A density range of 45 kg/m^2^ indicates crowding, which is associated with a significant decrease in body weight (BW) and bursa weight, feed consumption and conversion, flock uniformity, leg health, and increased frequencies of tibial dyschondroplasia, gait scores, carcass bruising, and scratching, and may lead to oxidative stress [6, 7]. Oxidative damage is a significant concern in poultry production. It occurs when exogenous or endogenous reactive oxygen species (ROS), which are associated with free radical formation and oxidative stress, overwhelm the antioxidant capacity of cells and extracellular spaces, thereby disrupting the redox balance of cells by influencing enzyme activation, signal transduction, and gene expression [8]. Changes in blood parameters have also been demonstrated under oxidative stress conditions, enhanced malondialdehyde (MDA) generation, and in association with reduced antioxidant enzyme activity [6].

White mulberry (*Morus alba* Linn.) is a plant with high nutritive value but a low cost that is widely found in Thailand. Efforts in Thailand are usually focused on identifying and using local trees and shrubs for livestock feeding. Mulberry leaf are considered a high-quality forage plant resource because of their high crude protein content (22%–29.8%), balanced amino acid composition, vitamins, and other bioactive substances (including flavonoids, polysaccharides, superoxide dismutase (SOD), sitosterol, isoquercitrin, r-amino acids, and 1-deoxynojirimycin) [8, 9]. They have various biological properties, including antioxidant, antimicrobial, glucosidase inhibitory, antihyperlipidemic, and antiatherosclerotic effects [10, 11].

Although researchers reported that adding a mulberry leaf to the diet of poultry significantly increased feed intake, egg production, and stimulated animal responses that may be related equally to the mulberry leaf providing phenolic compounds and functioning as a source of protein [11–13], the studies that focused on the relationship between stress, stock densities, and diet, including the mulberry leaf, are limited.

Therefore, this study was conducted to evaluate the impact of different stocking densities with or without mulberry leaf supplementation on growth performance, physiological stress, immune response, and changes in the antioxidant status in broilers.

## Materials and Methods

### Ethical approval

This study was approved by the Animal Ethics Committee for Use and Care of Kalasin University (Approval number: KSU17/2559).

### Study period and location

This study was conducted from August to December 2016. The study was conducted at the Institute of Veterinary Technology, Agricultural Technology, Kalasin University.

### Chemicals

Bovine serum albumin (BSA), 5,5-dithiobis-(2-nitrobenzoic acid) (DTNB), glutathione reductase, oxidized glutathione (GSSG), reduced glutathione (GSH), nitrotetrazolium blue chloride (NBT), reduced *β*-nicotinamide adenine dinucleotide phosphate (NADPH), standard malondialdehyde (MDA), standard superoxide dismutase (SOD) from bovine erythrocytes, 4-vinylpyridine (4-VP), and xanthine oxidase were purchased from Sigma Aldrich (St. Louis, MO, USA). Hydrogen peroxide (H_2_O_2_) was purchased from Thermo Fisher Scientific (Leicestershire, UK). 2-Thiobarbituric acid (TBA) was purchased by Fluka Chemika Co. (Steinheim, Switzerland). Trizol^®^ was purchased from Invitrogen^®^ (Carlsbad, CA, USA).

### Birds, diets, and housing conditions

Three-week-old male broilers (Cobb) were purchased from Charoen Pokphand Group (CO.), Thailand. The experimental facility was solid-sided. The temperature and light were both controlled. Ventilation consisted of fans producing positive pressure in the house. Birds were kept in a limited area with a wire partition (0.5 × 0.5 m^2^) at the university experimental farm in a room equipped with temperature control (25−30°C) and relative humidity control (around 50%). The light regimen was 11 h of continuous light per day. The birds were allowed free feeding with a commercial diet (20% protein, 4% fat, 5% fiber, and 11% moisture mix, CP911^®^, Charoen Pokphand Foods Company, Bangkok, Thailand) and water consumption *ad libitum* for all experimental treatments.

**Figure-1:**
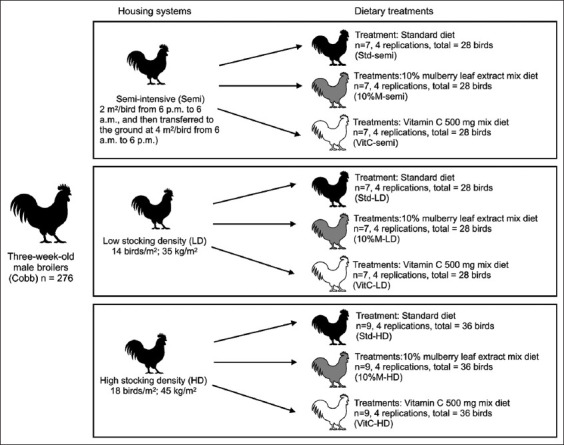
Experimental treatments of broilers. Three-week-old male broilers (Cobb) (n = 276) were divided into three housing classes including semi-intensive, low stocking density, and high stocking density. Each housing class was treated with three diets including standard diet, 10% mulberry leaf extract mix diet, and vitamin C 500 mg mix diet. Each experimental treatment was performed in four replicates.

The birds with similar BW (222.41 ± 24.63 g; mean ± standard deviation [SD]) were randomly selected. There were a total of 276 birds assigned to three classes (semi-intensive, low, and high). They received a standard diet (STD), a 10% mulberry leaf extract mix diet, or vitamin C supplementation [14–16], that is, a total of nine experimental treatments consisting of four replicates as follows (Figure-1) [17–19]:

1. Semi-intensive (broilers were raised in a house, i.e., a stall with windows (the space ratio of window to floor was 1:15 in the deep litter with free access to a grass-covered open-air run, 2 m^2^/bird from 6 pm to 6 am, and then transferred to the ground at 4 m^2^/bird from 6 am to 6 pm), with an STD (STD-semi) (n = 7);
2. Low stocking density (14 birds/m^2^; 35 kg/m^2^) with a STD (STD-LD) (n = 7);
3. High stocking density (18 birds/m^2^; 45 kg/m^2^) with a STD (STD-HD) (n = 9);
4. Semi-intensive with a 10% mulberry leaf extract mix diet (10% M-semi) (n = 7);
5. Low stocking density with a 10% mulberry leaf extract mix diet (10% M-LD) (n = 7);
6. High stocking density with a 10% mulberry leaf extract mix diet (10% M-HD) (n = 9);
7. Semi-intensive with a vitamin C 500 mg (Sigma Chemical Co. [St. Louis, MO, USA]) mix diet (vitamin C-semi) (n = 7);
8. Low stocking density with a vitamin C 500 mg mix diet (vitamin C-LD) (n = 7);
9. High stocking density with a vitamin C 500 mg mix diet (vitamin C-HD) (n=9).

Growth performance parameters, including food intake (FI), BW, average daily gain (ADG), and feed conversion ratio (FCR), were monitored on a daily and cumulative total basis for 4 weeks. Blood samples from all the birds were drawn from a wing vein and then transferred to chilled tubes containing ethylenediaminetetraacetic acid (EDTA) as an anticoagulant. Plasma was obtained from the blood by centrifugation at 400× *g* for 10 min at 4°C and was then stored at −20°C for further analysis at the Hematology Laboratory, Faculty of Veterinary Medicine, Khon Kaen University, Thailand.

### Preparation of mulberry leaf extract

Mulberry leaf were collected from the agricultural farm in Kalasin University, Kalasin Province, Thailand, at 16° 38’ 6.8316’’ N, 103° 46’ 18.732’’ E. The specimens were kindly identified by Professor Khwanruan Naksuwankul (Ph.D.) from the Faculty of Science, Mahasarakham University, according to the Flora of Thailand manual. The specimen voucher was deposited at the Natural Medicinal Mushroom Museum, Faculty of Science, Mahasarakham University (code number MSUT7709). Before being applied to the feed, the leaf were dried for 3 days at 25°C and ground into a fine powder (particle size, approximately 1 mm). The mulberry leaf powder was weighed and mixed with distilled water (1:10 ratio) for an hour at 95°C. After filtration (Advantec No. 1, Tokyo, Japan), the mulberry leaf extract solution was stored at −20°C for further analysis.

### Phytochemical screening of mulberry leaf extract

The biologically active chemicals, including alkaloids, phenolics, flavonoids, anthraquinone, coumarin, saponins, tannins, terpenoids, steroids, and glycosides, were screened using the previously described standard procedures. The total phenolic content (TPC) and total flavonoid content (TFC) of the crude extract were analyzed using gallic acid and quercetin as standard compounds. The TPC of the extract was determined using the Folin–Ciocalteu method [20]. The TFC of the crude extract was determined using the aluminum chloride colorimetric method. The TPC and TFC were analyzed using the calibration curves to calculate the concentrations in mg gallic acid equivalent (GAE)/g crude extract and mg quercetin equivalent (QE)/g crude extract, respectively [20].

### Determination of antioxidant enzyme activity

The blood samples were extracted using chloroform and ethanol, and the supernatant was then collected. The supernatant was mixed with xanthine, EDTA, Na_2_CO_3_, nitrotetrazolium blue chloride, and bovine serum albumin, and xanthine oxidase was added as required. For comparison, the same procedure was used with the bovine copper-zinc-SOD standard. CuCl_2_ was used to stop each reaction after 20 min of incubation at 25°C. The level of SOD activity was determined by the inhibition of formazan formation in the supernatant and the SOD standard with formazan was determined from its absorbance at 550 nm. The catalase (CAT) activity was measured as follows: The blood sample was incubated in a hydrogen peroxide (H_2_O_2_) substrate for 1 min at 37°C before the reaction was stopped with ammonium molybdate. The concentration of the yellow complex formed was measured at a wavelength of 405 nm and compared to the CAT standard.

The sample homogenate, 0.02 mM EDTA, 8.26 mM, sodium azide, and 2.48 mM sodium phosphate buffer (pH 7.4) were mixed and incubated for 10 min at 30°C. Glutathione (GSH) was then applied at a concentration of 1.24 mM H_2_O_2_ (SSA); and 3.31% (w/v) sulfosalicylic acid was used to initiate the reaction, which was stopped by adding SSA. The reaction mixture was centrifuged at 1900× *g* for 15 min. The activity of glutathione peroxidase (GPx) was measured using the supernatant [21, 22]**.**

### Measurement of GSH content

The blood samples were deproteinized with SSA and centrifuged at 10,000× *g* at 4°C for 10 min after being held at 2°C−8°C for 10 min. The reaction mixture [e.g., EDTA, potassium phosphate buffer (pH 7.0), GSH reductase, 5,5′-dithiobis-(2-nitrobenzoic acid), and nicotinamide adenine dinucleotide phosphate] was mixed with the supernatants to determine the total GSH. A spectrophotometer was used to calculate the absorbance of the thiol anions at a wavelength of 405 nm every 60 s for 5 min.

The total GSH content was calculated by comparing the sample’s A405/min (slope) to that of the GSH standard. The GSH content was calculated by subtracting the total GSH content from the GSSG, that is, a comparison of 4-vinyl pyridine (4-VP) incubation with an aliquot of the sample supernatant and the GSSG standard incubation at room temperature (25°C) for 60 min. The procedure for determining the GSSG content was similar to that for determining the GSH content [22, 23].

### Determination of lipid peroxidation

A thiobarbituric acid (TBA) assay was used to assess lipid peroxidation. The reaction mixture (e.g., trichloroacetic acid, acetic acid, and 2-TBA) was added after the blood sample. The MDA standard was incubated for 1 h at 37°C. For 15 min, the samples were boiled. The TBA reactive species (or TBARS) were measured using a spectrofluorometer with an emission wavelength of 551 nm and an excitation wavelength of 528 nm [22].

### Statistical analysis

The experimental results were analyzed using a two-way analysis of variance with Tukey’s multiple comparisons test by Prism version 9 (GraphPad Soft Inc., La Jolla, CA, USA). The results are expressed as the mean ± (SD). Statistical significance was inferred at p ≤ 0.05 [16, 24].

## Results

### Phytochemical screening of mulberry leaf extract

The phytochemical screening of the mulberry leaf extract was analyzed. The alkaloids, phenolics, flavonoids, coumarin, tannins, and terpenoids were present in the mulberry leaf extract. The TPC calculated from the calibration curve (R^2^ = 0.998) was 61.075 ± 4.798 mg GAE/g for the crude extract, and the TFC (R^2^ = 0.987) was 207.255 ± 3.396 mg QE/g for the crude extract (Table-1).

**Table-1:** Phytochemical screening of mulberry leaf extract.

Phytochemical screening tests	
Alkaloids	Positive
Phenolics	Positive
Flavonoids	Positive
Anthraquinones	Not detected
Coumarin	Positive
Saponins	Not detected
Tannins	Positive
Terpenoids	Positive
Steroids	Not detected
Glycosides	Not detected
Quantitative analysis	
Total phenolic content	61.075 ± 4.798 mg GAE/g crude extract
Total flavonoid content	207.255 ± 3.396 mg QE/g crude extract

GAE=Gallic acid equivalent, QE=Quercetin equivalent

### Growth performances

**Figure-2:**
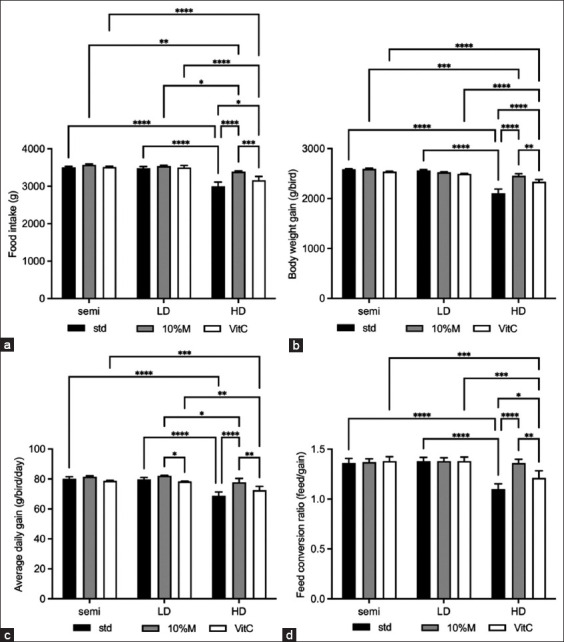
Growth performance of broilers fed with mulberry and vitamin C under three raising systems. (a) Feed intake, (b) body weight gain, (c) average daily gain, and (d) feed conversion ratio. The data are presented as the mean ± standard deviation. Different symbols indicate a significant difference among different precipitation levels (*p ≤ 0.05, ** p ≤ 0.01, *** p ≤ 0.001, and **** p ≤ 0.0001).

The mortality rate of the birds was zero. Throughout the study period, all the birds remained healthy. As regard the growth performance in the semi-intensive (Semi) and low-density (LD) classes, there was no statistical difference in the performance, but significance was observed in the high-density (HD) class (the performances were extremely reduced; all of FI, BW, ADG, and FCR; p ≤ 0.001). The adding 10% mulberry leaf extract (10% M) resulted in no significant change in the performance in the Semi or LD classes; however, the performances of the HD class significantly improved (the value was close to that of the Semi or LD class). Adding vitamin C to each class had higher FI (p ≤ 0.05), BW (p ≤ 0.01), and FCR (p ≤ 0.05) than those in the STD-HD group but lesser in 10% M-HD group (p ≤ 0.001) that is illustrated in Figure-2.

### Hematological parameters

**Figure-3:**
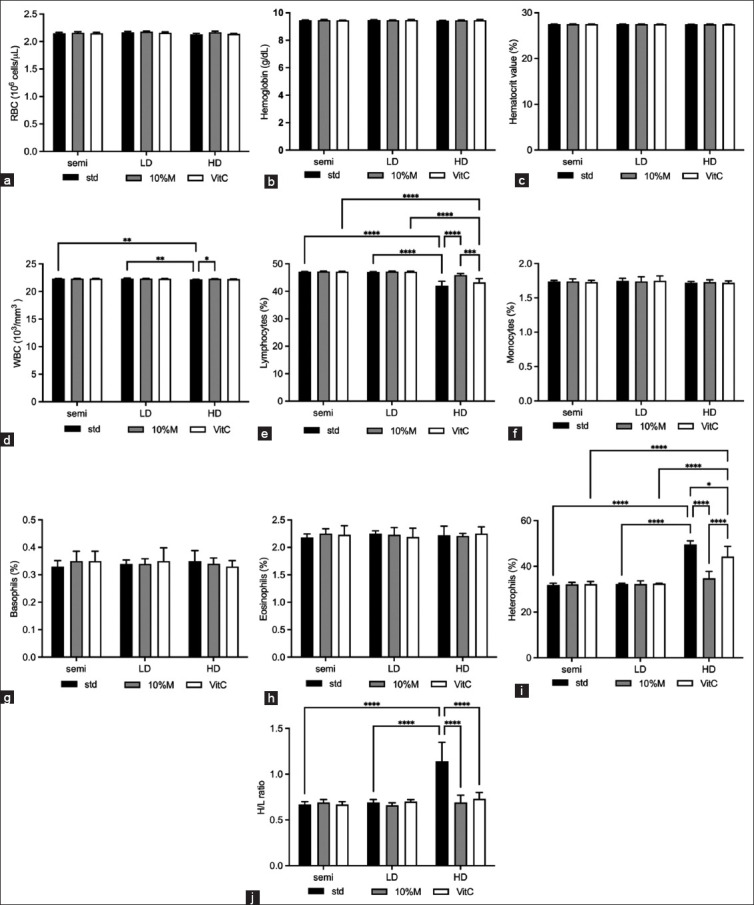
Hematology parameters of broilers with mulberry and vitamin C under three raising systems. (a) red blood cells (RBCs), (b) hemoglobin, (c) hematocrit value, (d) white blood cells (WBCs), (e) lymphocytes, (f) monocytes, (g) basophils, (h) eosinophils, (i) heterophils, and (j) heterophile-to-lymphocyte (H/L) ratio. The data are presented as the mean ± standard deviation. Different symbols indicate a significant difference among different precipitation levels (*p ≤ 0.05, **p ≤ 0.01, ***p ≤ 0.001, and ****p ≤ 0.0001). Standard levels of each parameter including, RBCs = 2.15 ± 0.06 106/μL, hemoglobin = 9.50 ± 0.38 g/DL, hematocrit value = 28.90 ± 1.98%, WBCs = 22.50 ± 0.85 103/mm3, lymphocytes = 48.40 ± 0.85 %, monocytes = 1.70 ± 0.35%, basophils = 0.81 ± 0.36%, eosinophils = 2.27 ± 0.39%, heterophils = 32.0 ± 1.89%, and H/L ratio = 0.58 ± 0.20 [25–27].

Figure-3 shows the red and white blood cell parameters [25–27]. There was no statistical difference for any parameter from either the red blood in the semi or LD classes. Similarly, only the heterophile-to-lymphocyte (H/L) ratio of the white blood cells (corresponding to lymphocytes and heterophile) in the HD class was significantly increased (with low values of lymphocyte and high values of heterophile). Adding either 10% M or vitamin C had no significant impact on H/L ratio of the semi or LD classes; however, in the HD class, 10% M group had significantly improved (the H/L ratio was reduced to close to that of the semi and LD classes) better than vitamin C group (p ≤ 0.001).

### Antioxidant profiles

**Figure-4:**
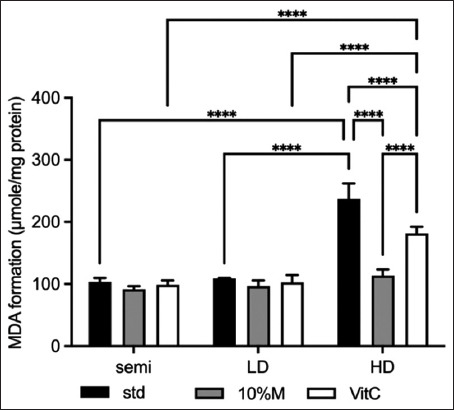
The level of lipid peroxidation of broilers fed with mulberry under three raising systems. The data are presented as the mean ± standard deviation. Different symbols indicate a significant difference among different precipitation levels (*p ≤ 0.05, **p ≤ 0.01, ***p ≤ 0.001, and ****p ≤ 0.0001).

Figure-4 shows the level of lipid peroxidation, which was used to indicate oxidative stress. There was no statistical difference (STD, 10% M, and vitamin C) between the semi and LD classes. In the HD class, there was a rapid and significant increase in the lipid peroxidation level (MDA) for the STD group. Although adding vitamin C and 10% M were able to significantly reduce the MDA, interestingly, 10% M was able to significantly lower the lipid peroxidation level, such that there was no significant difference among all classes.

**Figure-5:**
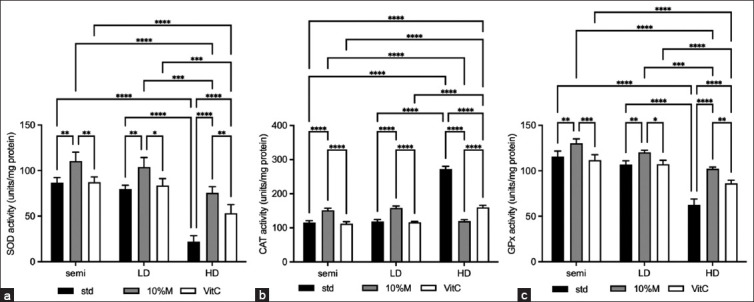
Antioxidant enzyme activity of broilers fed with mulberry under three raising systems. (a) superoxide dismutase activity, (b) catalase activity, and (c) glutathione peroxidase activity. The data are presented as the mean ± standard deviation. Different symbols indicate a significant difference among different precipitation levels (*p ≤ 0.05, **p ≤ 0.01, and ***p ≤ 0.001).

Figure-5 shows the three metrics of antioxidant enzyme activity: SOD, CAT, and GPx. In the STD, the SOD activity in HD was substantially decreased but no statistical difference was observed in the semi or LD classes. While adding vitamin C and 10% M were able to significantly increase the activity, adding 10% M to the HD class brought the activity up to the STD group levels in the semi and LD classes. It can be observed from Figure-2c that the trends of GPx activities were similar to those of SOD activities. In Figure-2b, only the semi and LD classes exhibited the same trends (STD, 10% M, and vitamin C) for SOD and GPx activities as for CAT activity. In the HD class, the activity of the STD group was extremely high, while adding 10% M was able to lower the activity to close that of the semi and LD classes (STD).

## Discussion

The rearing method is one of the most critical non-genetic factors that influence productivity and health in broiler chickens [19]. In Thailand, there are various types of poultry production systems, the most common of which are caged housing (or intense systems) and semi-intensive systems (Semi). Semi-intensive systems are an alternative method of rearing broilers in which the birds are raised in a poultry house and given free access to a grazing area during the day [28].

In the main point, this study investigated the birds’ growth performance, blood biochemistry, and antioxidant status at various population densities to improve our understanding of crowding stress in birds.

In the growth performance, there were no statistically significant interactions between the semi and LD classes. These findings support previous research that found a higher proportion of comfort behaviors in the free-range system and low stocking densities (10−14 birds/m^2^) [18, 19], while in the HD class (18 birds/m^2^) had a low feed intake, BW gain, ADG, and FCR, and these growth performance metrics were lower than those of the other classes (semi and LD). Several investigations indicated a decrease in feed intake due to overcrowding, thereby corroborating these findings [14, 15].

According to Sugiharto *et al*. [29], high stocking densities hinder the mobility of birds in a particular area, causing them to fight for access to feeders and drinkers. In addition, the reduction in growth performance in the HD class could be linked to a rise in temperature with a decrease in air circulation at the bird level [24]. According to several studies, HD systems increase stress, which increases corticosterone release, limiting glucose utilization, and therefore, protein accretion-based growth is reduced [25, 30]. The HD systems restrict growth and access to feed, potentially resulting in nutritional shortfalls and high-energy expenditure, exacerbating stress, and increasing the metabolic rate, negatively impacting growth performance [24].

Moreover, HD systems have an impact not only on growth but also on immunological characteristics. This result showed the reduction of lymphocytes while the number of heterophils was increased. The high H/L ratio and high corticosterone levels imply stress [15, 26]. These findings are supported by the results of Nasr *et al*. [24], who found that factors such as glucocorticoid release cause lymphocytes in lymphoid tissues to deteriorate and promote the bone marrow to synthesize heterophils [27].

As regard the antioxidant profile, the HD system induced oxidative stress in broilers, increasing MDA while decreasing SOD and GPx activity in the serum. A previous study on the negative effects of high broiler stocking density agree with our findings [31]. This may be due to increased competition among birds in a limited space, creating metabolic abnormalities. As a result, the process of inducing stress, triggering increased lipid peroxidation and high ROS production, boosting oxidative damage, and creating MDA could potentially reduce antioxidant enzyme activity [32, 33]. Higher MDA levels in HD raised birds are consistent with the findings of Mohammed *et al*. [34], who found that crowding increased oxidative damage and resulted in MDA generation.

Because MDA is one of the end products of lipid peroxidation, the development of oxidative damage as a result of increased free radical generation could be detected by measuring the MDA serum concentration [35]. SOD, CAT, and GPx are antioxidant enzymes that are the first line of defense against antioxidant reactions [32]. Corresponding to the results concerning the effect of antioxidant enzyme activity in this study, SOD acts as the first line of defense against the deleterious effects of oxyradicals in cells by catalyzing the dismutation of the endogenous cytotoxic superoxide radical into H_2_O_2_, which is then detoxified into water and oxygen by the joint action of GPx and CAT [36]. This finding was conversely in the semi and LD classes; there were no statistically significant interactions between them [24, 34].

In the second point, mulberry leaf (a traditional Thai plant) have long been considered high in nutrients, notably crude protein, and are known to provide amino acid balance and are highly digestible [37]. The study of Has *et al*. [38] showed that 10% mulberry leaf improved rumen liquor fermentation in broilers. Similarly, the study by Ding *et al*. [9] showed that 9% mulberry leaf could improve the meat quality and muscles of boilers without affecting the growth performance. The phytochemical screening of mulberry leaf extract revealed alkaloids, phenolics, flavonoids, coumarin, tannins, and terpenoids. These results are similar to those published by Wang *et al*. [12].

Bioactive compounds found in the leaf include jasmonic acid, anthocyanins, flavonoids, stilbene, and terpenoids. The effect of these compounds involves antibacterial, antipyretic, anticancer, antioxidation, hypoglycemic, serum lipid lowering, and metabolism improving characteristics [32, 39]. The mulberry leaf is highly palatable and easily digestible (70%−90%) for herbivores and can be fed to monogastric animals. The crude protein content of mulberry leaf ranges from 15%−28% for geese, beef cattle, sheep, pigs, laying hens, and broilers [12, 40, 41].

The considerable improvement in growth performance shows that mulberry had a positive effect on oxidative status, which explains why the FCR was enhanced, but vitamin C was not. In this study, the performance of the HD class was significantly improved by adding 10% M, which corresponds to the report by Hao *et al*. [42]. An investigation into mulberry leaf as a high-quality forage showed them to have high crude protein (22%–29.8%) and tannin (anti-nutritional factor) contents. The excessive addition of mulberry leaf affects livestock and poultry production performance and health. In ruminants, mulberry leaf are an unconventional feed resource and are mainly used for sheep and cattle, but there is little research on using them with broilers [7, 43]. According to Lin *et al*. [8], various herbs and their extracts can help the digestive tract by enhancing the activity of digestive enzymes in the gastric mucosa, potentially improving growth performance.

In HD class with 10% M, MDA concentrations were significantly reduced, while antioxidant enzyme activity in the serum remained high. With vitamin C, the concentration was similarly reduced, but to a lesser extent. The reduction in lipid peroxidation observed after the addition of 10% mulberry leaf reflects their free radical scavenging activity and antioxidant capacity, as evidenced by its *in vitro* antioxidant properties. About 10% M increased antioxidant enzyme levels (SOD, CAT, and GPx) in the semi and LD groups. Mulberry leaf improved the overall antioxidant capacity, and SOD and immune globulin levels, according to Hassan *et al*. [39]. Adding 10% M in the HD class potentially improved the activities to levels close to those of the STD for the semi and LD classes because of the increase in the broilers’ ability to scavenge free radicals and antioxidant capacity activation.

In addition, vitamin C was able to reduce the MDA and improve antioxidant activities, but, as compared to 10% M, the ability was lower. Studies have also shown that flavonoids can boost antioxidant capacity, increase nonspecific immunity, and reduce oxidative stress by raising SOD, CAT, and GPx activity while lowering MDA levels. The effect is mainly attributed to the ability of flavonoids to act as reducing agents and hydrogen donors to neutralize ROS and eliminate H_2_O_2_ and superoxide ions. Mulberry leafhave dual functionality in reducing oxidative stress, according to the findings of numerous studies: (1) by directly interacting with ROS and (2) by increasing the activity of antioxidant enzymes [8, 44].

## Conclusion

It can be concluded that growth performance was decreased in HD class. The high H/L ratio and corticosterone levels that imply the stress. As regard the antioxidant profile, the HD system induced oxidative stress in broilers, increasing MDA while decreasing antioxidant activity in the serum. Mulberry leaf extract was found to be beneficial in normalizing the hematological parameters of birds under oxidative stress based on the findings of this study. In addition, it can aid in reducing free radicals and enhancing antioxidant enzyme function. As a result, giving mulberry leaf extract to broiler birds increases their efficiency, allowing them to create higher-quality products.

## Authors’ Contributions

CS and NS: Conceived and designed the study. CS: Performed the study. CS and NS: Analyzed the data. CS and NS: Contributed reagents/materials/analysis tools. CS and NS: Wrote the manuscript. All authors have read and approved the final manuscript.
